# Non-adherence, medication beliefs and symptom burden among patients receiving hemodialysis -a cross-sectional study

**DOI:** 10.1186/s12882-023-03371-3

**Published:** 2023-10-27

**Authors:** Trine Mechta Nielsen, Trine Marott, Mads Hornum, Bo Feldt-Rasmussen, Thomas Kallemose, Thordis Thomsen

**Affiliations:** 1 Department of Nephrology, Centre for Cancer and Organ Diseases, Copenhagen University Hospital - Rigshospitalet, Copenhagen, Denmark; 2 Department of Nephrology, Copenhagen University Hospital - Herlev, Copenhagen, Denmark; 3 Department of Clinical Medicine, Faculty of Health and Medical Sciences, University of Copenhagen, Copenhagen, Denmark; 4 Department of Clinical Research, Copenhagen University Hospital – Amager and Hvidovre, Copenhagen, Denmark; 5 Herlev Acute, Critical and Emergency Science Unit - Herlev-ACES, Department of Anesthesiology, Copenhagen University Hospital -Herlev, Copenhagen, Denmark

**Keywords:** Beliefs about medication, Cross-sectional study, Hemodialysis, Medication adherence, Symptom management

## Abstract

**Background:**

Non-adherence to medication is a common and complex issue faced by individuals undergoing hemodialysis (HD). However, more knowledge is needed about modifiable factors influence on non-adherence. This study investigated the prevalence of non-adherence, medication beliefs and symptom burden and severity among patients receiving HD in Denmark. Associations between non-adherence, medications beliefs and symptom burden and severity were also explored.

**Method:**

A cross-sectional questionnaire-based multisite study, including 385 participants. We involved patient research consultants in the study design process and the following instruments were included: Medication Adherence Report Scale, Beliefs about Medication Questionnaire and Dialysis Symptom Index. Logistic regression analysis was performed.

**Results:**

The prevalence of non-adherence was 32% (95% CI 27–37%) using a 23-point-cut-off. Just over one third reported being concerned about medication One third also believed physicians to overprescribe medication, which was associated with 18% increased odds of non-adherence. Symptom burden and severity were high, with the most common symptoms being tiredness/ lack of energy, itching, dry mouth, trouble sleeping and difficulties concentrating. A high symptom burden and/or symptom severity score was associated with an increased odd of non-adherence.

**Conclusion:**

The study found significant associations between non-adherence and, beliefs about overuse, symptom burden and symptom severity. Our results suggest health care professionals (HCP) should prioritize discussion about medication adherence with patients with focus on addressing patient-HCP relationship, and patients’ symptom experience. Future research is recommended to explore the effects of systematically using validated adherence measures in clinical practice on medication adherence, patient-HCP communication and trust. Additionally, studies are warranted to further investigate the relationship between symptom experience and adherence in this population.

**Trial registration:**

NCT03897231.

**Supplementary Information:**

The online version contains supplementary material available at 10.1186/s12882-023-03371-3.

## Introduction

Medication adherence is: “the extent to which a person’s behavior of taking medication corresponds with agreed recommendations from a healthcare provider” [1]. Patients with End Stage Kidney Disease (ESKD) undergoing hemodialysis (HD) cope with a demanding medication regime, that includes multiple medications, health care appointments with medication regulation, renewal of prescriptions and continuously having to adapt their medication behavior to the prescribed regime [2, 3]. Not surprisingly, non-adherence to medication is prevalent among this patient population, with estimates ranging from 12–98% [4, 5]. Non-adherence increases the risk of disease progression, rising health care costs due to hospitalization, and premature death [5–7].

The issue of non-adherence among patients in HD is multifaceted and several demographic factors have been suggested to be associated with non-adherence including young age, non-Caucasian ethnicity, female gender, living alone and low educational level. Clinical factors include longevity of HD, concurrent diseases and high pill burden [5]. Other factors are modifiable, meaning they can be changed or addressed to improve adherence. A recent metanalysis found that negative beliefs about medication among a wide range of chronic conditions, were associated with non-adherence [8]. This indicates that the motivation of patients to initiate and adhere to treatment is shaped by their own beliefs and preferences [3]. Cultural variations have been found across countries. Nevertheless, non-adherence among patients undergoing HD in Scandinavia is sparsely researched [8]. The UCSF Symptom Management Model describes a complex and two-way connection between symptom experience, patients’ beliefs and adherence [9–11]. Patient-reported symptoms have been consistently associated to medication adherence in patients living with HIV [12]. Thus, studies have found that a high quantity of symptoms, also called symptom burden, was associated with poorer medication adherence [9, 12]. Patients in HD report a high prevalence of physical and emotional symptoms throughout the literature [13–16]. It is therefore reasonable to hypothesize that symptom burden also impacts adherence in the HD population. However, research investigating association between symptom burden and non-adherence in HD settings is limited.

### Aims

The aims of this study were therefore to investigate 1) the prevalence of non-adherence 2) patients’ beliefs about medication 3) symptom burden and severity and 4) associations between beliefs about medication, symptom burden and severity and non-adherence in patients receiving HD.

## Materials and methods

A multi-site cross-sectional study was conducted from April 2019 – December 2020 at four University Hospitals in the Capital Region of Denmark, involving patients with ESKD receiving HD treatment in a hospital-based outpatient center.

### Patient involvement

We involved patients in the study design process to increase patient recruitment and obtain relevant knowledge applicable to the HD population. We built on an already well-established collaboration with one patient research consultant (PRC) [17]. Three additional PRCs were recruited by this consultant. All had current or previous experience with HD treatment and represented diverse ages, gender and education.

We held two workshops. In the first workshop TMN introduced a sample of possible instruments related to medication adherence and questions for collecting basic demographic data. The PRCs were asked to identify the variables that mattered the most to them in relation to medicine taking. This resulted in removal of the Kidney Disease Quality of Life questionnaire (KDQOL) and the Hospital Anxiety and Depression Scale (HADS) and to the inclusion of the Medication Adherence Report Scale (MARS), Beliefs about Medication Questionnaire (BMQ) and the Dialysis Symptom Index (DSI). In the following workshop, the PRCs commented on the study information leaflet. Additionally, they pilot-tested and evaluated the clarity, difficulty, and appropriateness of the Danish translation of MARS and DSI. The preliminary version of the entire survey was subsequently discussed via phone and email, and the final version of the questionnaire prepared.

### Procedure

Participants who were ≥ 18 years of age, who had been undergoing HD treatment ≥ 3 month at a hospital-based outpatient HD Center were approached by their allocated nurses during dialysis and informed about the study. Participants who were judged by the health care professional (HCP) as not able to answer questions due to cognitive impairment, psychiatric disorder or not being able to understand or speak Danish were excluded. Those who wished to participate provided informed consent and completed a paper-based questionnaire. Patients who had visual or physical impairments or difficulty reading Danish were interviewed using a structured interview approach.

### Sample size

Sample size was determined based on the precision of the non-adherence prevalence estimate. With an expected 50% prevalence of non- adherence and a 95% confidence interval (CI) with a 5% ± margin of error, 385 participants were required.

### Data collection

The questionnaire consisted of demographic and clinical data and the following instruments; MARS to assess medicine taking behavior [18], BMQ to assess medication beliefs [19] and DSI to measure physical and emotional symptoms and their severity [13]. Please refer to Table 1 to get a full overview of the instruments applied. Before collecting data MARS and DSI underwent translation from English into Danish followed a three-step process inspired by the European Organization for Research and Treatment of Cancer (EORCT) translation procedure [20].

**Table 1:** Full overview of instruments applied

Instrument	Scoring system	Validity
Medication Adherence Report Scale (MARS) [18]	MARS assesses medicine taking behavior with 5 items rated on a 5-point Likert scale ranging from always (1 point) to never (5 points). Scores for each of the five items are aggregated to give the final score (5 to 25 points), with higher scores indicating better adherence. The cut-off score for non-adherence is not standardized [21], but commonly used values are < = 25 [22, 23] and < = 23 [21, 24, 25]. We defined non-adherence as a total score less than 23, considering the challenge of perfect adherence. Nevertheless, results of both cut-off scores, as previously described in the protocol for this study	Widely used within the patient population and has demonstrated acceptable internal consistency. MARS has not undergone validation in Danish context
Beliefs about Medicines Questionnaire (BMQ) [19]	The BMQ assesses patients’ medication beliefs. It includes 18 items rated on a 5-point Likert scale going from strongly disagree to strongly agree. BMQ consists of two categories, BMQ General and BMQ Specific. BMQ general measures beliefs about harm and overuse, with scores ranging from 4–20 (midpoint: 12). BMQ specific evaluates concerns and perceived necessity, with scores ranging from 5–25 (midpoint: 15). Higher scores indicate stronger beliefs about harm, overuse, concern and necessity. The necessity-concern differential score assesses the balance between concern and necessity ranging from -20 to 20. A positive score indicates that patients believe that the benefits of taking medication outweigh their concerns	Widely used within the patient population and has demonstrated acceptable validity and reliability
Dialysis symptom index (DSI) [13]	DSI measures physical and emotional symptoms and their severity. It assesses the prevalence and severity of 30 symptoms over the past week on a 5-point Likert scale going from “not at all bothersome” to “bothers very much.” The total number of reported symptoms reflects the overall symptom burden, ranging from 0–30. The severity score ranges from zero (no symptoms) to 150 (30 symptoms)	Widely used within the patient population and has demonstrated good validity and reliability. DSI has not undergone validation in Danish context

Demographic and clinical data included social security number, gender, age, country of birth, living arrangements, marital status, education, occupation, comorbidities, type of dialysis, longevity of HD, kidney transplantation history and daily pill burden. Daily pill burden was calculated based on the daily pill count and applied exclusively to oral medications, excluding medications prescribed pro nececsitate.

All data were entered into REDCap by a project nurse. Followed by a screening for typing errors and missing values. Based on the screening, our assumption was, that the missing values occurred randomly. Therefore, if more than two items were missing in MARS, BMQ and DSI, the missing items were replaced with the average of the other items.

### Confounders

Potential confounders included gender, age, country of birth, living arrangements, marital status, comorbidities, longevity of HD and daily pill burden. The confounders were selected based on previous research indicating their significant association with non-adherence [5]. Due to symptoms being associated to medication adherence in other patient populations [12] both symptom burden and symptoms severity were selected as potential confounders to necessity, concern, harm and overuse in the BMQ.

### Statistical analysis

Continuous data are presented as means with standard deviations (SD) or medians with interquartile ranges (IQR), depending on distribution of data. Categorical data are presented as count with percentages. The results for individual BMQ sub-scales are presented as percentages over midscale scores and as medians with IQRs. The DSI scores are presented as medians and IQRs. Prevalence estimates are presented as percentages with 95% confidence intervals (CI). Associations between independent variables (necessity, concern, harm, overuse, symptom severity score, symptom burden score) and our dependent variable (non-adherence), were analyzed using logistic regression modelling. Each variable was fitted into an unadjusted model and models controlling for possible effects of confounders. Confounders were evaluated individually, by comparison of the unadjusted independent variable estimate with the independent variable estimate in the models adjusting for each confounder separately. The final adjusted regression model included the three confounders that had the greatest change on the independent variable estimate (please see supplementary material for an overview of the analyses conducted). Model estimates were presented as odds ratios (OR) with 95% Confidence Intervals (CI) and *P*-values. *P*-values less than 0.05 were considered significant. We used Hosmer–Lemeshow test to evaluate goodness-of-fit and the Bonferroni correction to account for multiple testing by upscaling *p*-values with the number of tests performed. Statistical analysis was performed using IBM SPSS Statistics V.25.0

## Results

**Fig. 1:**
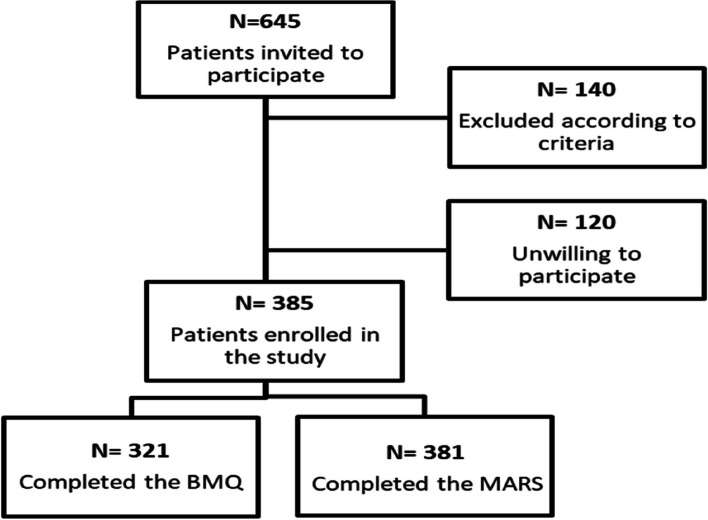
Patient enrollment flowchart

A total of 385 patients were enrolled in the study (Fig. 1). The majority (two thirds) were male patients, elderly of age, and had attended HD treatment for > 2 years. Nearly one third had a level of education equivalent to university. Please see Table 2 for patient characteristics.

**Table 2:** Characteristics of participants

Demographic and clinical variables	Participants (*N* = 385)
**Age (median, IQR)**	67 years (18)
**Sex**	
• Male	66,5% (*n* = 256)
• Female	33,5% (*n* = 129)
**Country of birth**	
• Denmark	80,5% (*n* = 310)
• Europe	5,2% (*n* = 20)
• Other	11,7% (*n* = 45)
**Living alone**	48,8% (*n* = 188)
**Type of dialysis**	
• Center dialysis	88,8% (*n* = 342)
• Limited care	9,1% (*n* = 35)
• Self-care	2,1% (*n* = 8)
**Time in dialysis (median, IQR)**	27, 7 months (51)
**Completed education**	
• Primary school	54% (*n* = 208)
• Secondary school	11,9% (*n* = 46)
• University	29,1% (*n* = 112)
**Comorbidities**	
• Diabetes type 1	4,9% (*n* = 19)
• Diabetes type 2	29,9% (*n* = 115)
• Hypertension	42,9% (*n* = 165)
• COL	7,3% (*n* = 28)
• Eye disease	22,9% (*n* = 88)
• Current cancer disease	7,8% (*n* = 30)
**Kidney transplantation**	
• Previous TX	13,8% (*n* = 53)
• On waiting list	20,3% (*n* = 78)
**Daily pill burden (mean, SD)**	18,7 pills (7)
**Use of administering box**	73% (*n* = 282)

### Prevalence of non-adherence

Based on a cut-off score < 23 points in the MARS assessment, 32% (95% CI 27–37%) were identified as non-adherent to their prescribed medications. However, when the cut-off score was altered to < 25 points, the number of non-adherent participants increased to 73% (CI 69–77%). Forgetfulness was the most frequent reason for non-adherence, cited by 62% of participants. Altering the dose was the second most frequent reason, with 36% of participants reporting doing so rarely or often (Table 3).

**Table 3:** Results of MARS

**MARS**	**N**	**Mean + — SD**	**Median (IQR)**	**%**^a^
I forget to take my medicines	382	4,19 (0.75)	4 (1)	62
I alter the dose of my medicines	382	4.49 (0.77)	5 (1)	36
I stop taking my medicines for a while	381	4.81 (0.52)	5 (0)	14
I decide to miss out a dose of my medicines	382	4.65 (0.66)	5 (1)	25
I take less of my medicines than instructed	382	4.72 (0.64)	5 (0)	20

^a^Percentage of participants answering the question with rarely, sometimes or often

### Patients’ beliefs about medication

The BMQ revealed that while most participants believed medication to be necessary, a large proportion expressed concern about taking them. Although the majority did not consider medication to be harmful, 35% believed physicians overuse medication in general. The necessity and concern differential were 7, with 86% scoring positively, indicating a belief that the benefits of medication outweighed their concern. However, 14% of participants (*n* = 53) scored zero or below, suggesting a higher concern for medication than a sense of necessity (Table 4).

**Table 4:** Results of the BMQ

BMQ sub-scale	Valid cases	Estimates	Patients scoring above midscale %
**Necessity (median, IQR)**	375	21.0 (5.0)	92.6
• My health depends on my medicines		5.0 (1.0)	
• My life would be impossible without my medicines		4.0 (1.0)	
• Without my medicines I would be very ill		4.0 (1.0)	
• My health will depend on my medicines in the future		4.0 (1.0)	
• My medicines protect me from becoming worse		4.0 (1.0)	
**Concern (mean, SD)**	373	14.3 (4.4)	40.8
• Having to take my medicines worries me		3.0 (1.2)	
• I sometime worry about long-term effects of my medicines		3.3 (1.2)	
• My medicines are a mystery to me		2.6 (1.2)	
• My medicines disrupt my life		2.8 (1.2)	
• I sometimes worry about becoming too dependent on my medicines		2.6 (1.2)	
**Harm (mean, SD)**	373	9.9 (3.0)	18.2
• All medicines are poisons		2.5 (1.0)	
• Medicines do more harm than good		2.7 (1.0)	
• Most medicines are addictive		2.2 (0.9)	
• People who take medicines should stop their treatment for a while every now and again		2.4 (1.2)	
**Overuse (mean, SD)**	372	11.3 (3.1)	34.5
• Doctors use too many medicines		2.8 (1.0)	
• Natural remedies are safer than medicines		2.4 (1.0)	
• Doctors place too much trust in medicines		3.0 (1.0)	
• If doctors had more time with patients, they would prescribe fewer medicines		3.1 (1.0)	

### Prevalence and severity of physical and emotional symptoms

**Fig. 2:**
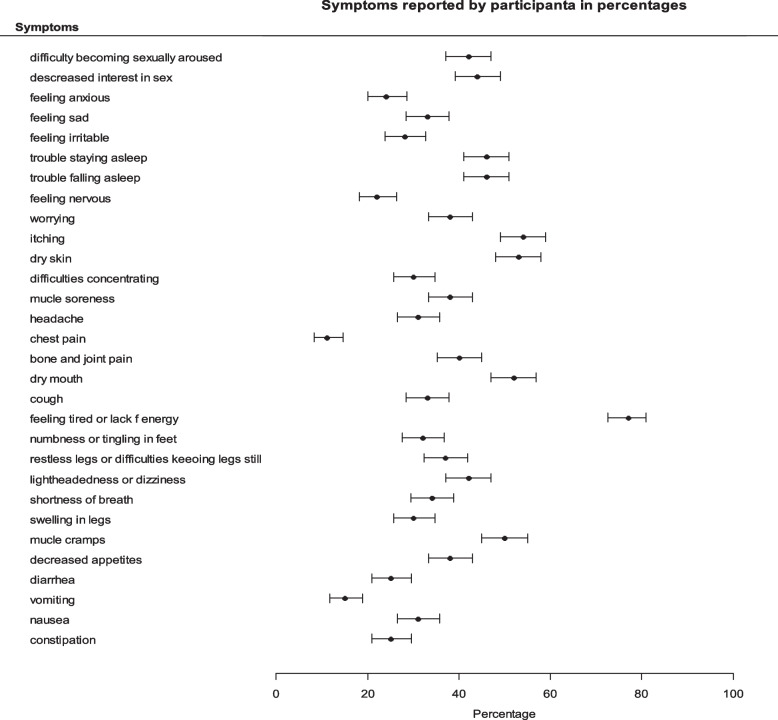
Symptoms reported by participants in percentage

The median symptom burden score was 10 (IQR 5–16) with a minimum score of 0 (*n* = 20) and maximum score of 30 (*n* = 1). Similarly, the median score for total symptom severity score was 30 (IQR 14–53), with the lowest reported score being 0 (*n* = 20) and the highest reported score being 106 (*n* = 1). The most prominent symptoms were feeling tired/lack of energy (77%), itching (54%), dry mouth (52%), lightheadedness/dizziness (42%), trouble falling asleep/staying asleep (46%) and difficulty concentrating (39%) (Fig. 2).

### Non-adherence and patients’ beliefs about medication

After adjusting for confounding variables, only beliefs about overuse (OR = 1.18, CI = 1.09–1.27) were significantly associated with non-adherence (Table 5).

**Table 5:** ORs of non-adherence

Non-adherence	Unadjusted OR, 95% CI, *P* value	Adjusted OR, 95% CI, *P* value
Necessity*	0.94, 0.88 to 1.00, 0.228	0.95, 0.87 to 1.03, 0.432
Concern*	1.08, 1.02 to 1.13, 0.020	1.08, 1.01 to 1.15, 0.12
Harm*	1.09, 1.01 to 1.17, 0.092	1.10, 1.01 to 1.21, 0.140
Overuse*	1.17, 1.09 to 1.26, < 0.001	1.18, 1.09 to 1.27, < **0.001**
Symptom severity**	1.02, 1.01 to 1.03, < 0.001	1.02, 1.01 to 1.03, < **0.001**
Symptom burden**	1.08, 1.05 to 1.12, < 0.001	1.09, 1.05 to 1.13, < **0.001**

^*^Necessity, concern, harm and overuse: P-value were upscaled with 4 in accordance with the Bonferroni correction

^**^Symptom severity and symptom burden: P-value were upscaled with 2 in accordance with the Bonferroni correction

### Non-adherence and patient experienced symptoms burden and severity

Symptom severity (OR = 1.02, CI = 1.01–1.03) and symptom burden score (OR = 1.09, CI = 1.05–1.13) were significantly associated with non-adherence (Table 5).

## Discussion

This study investigated the prevalence of non-adherence, patients’ beliefs about medication and the prevalence and severity of physical and emotional symptoms among Danish patients receiving HD. Potential associations between beliefs about medications, symptom burden- and severity and non-adherence were also investigated.

The prevalence of non-adherence was assessed to 32% using a 23-point cut-off in the MARS. However, when adjusting the cut-off to 25-points, non-adherence increased to 73%. Both results fall within the range of previously reported rates observed in other HD settings (ranging from 12 to 98%) [5]. The results also underscore the challenge posed by inconsistent definitions of non-adherence, contributing to the substantial variation in prevalence rates across studies. Surprisingly, 27% of participants reported in MARS that they never missed a dose, suggesting possible challenges with recall bias and over/under estimation when using self-report questionnaires to assess non-adherence [26, 27]. Nevertheless, self-report measures are valuable tools for evaluating medication adherence as they capture subjective experiences and perceptions [27]. Accordingly, our study emphasizes the relevance of using PROMs routinely in clinical practice, both to monitor adherence and as a starting point for HCPs to discuss and support patients in their medication taking efforts.

Most participants believed medication to be necessary, although over one third reported concerns about taking them. The majority did not consider medication to be harmful, but one third believed that physicians in general overused medication. These findings resonate with previous research [8, 26, 28]. Drangsholt et al. similarly found a high necessity score of 98% and a low concern score of 34% in a Norwegian HD population [28]. This similarity may be explained by shared cultural values [8]. Nevertheless, the findings illustrate the complex interplay between patients having to struggle with being dependent on medication and at the same time harboring concerns about taking them and the belief that medication is overprescribed by physicians.

Notably, our results revealed 18% increased odds when harboring the belief that physicians overuse medications. This aligns with a recent study among patients with asthma, showing a 40% decrease in adherence when believing physicians to overuse medication [29]. It has been suggested that the nature of the patient-HCP relationship, particularly in relation to miscommunication and mistrust, may be linked to this association [30]. Both miscommunication and mistrust have previously been reported by patients as barriers to adherence [31]. The absence of systematic screening of adherence and standardized approaches among physicians and nurses to supporting adherence may also contribute. Time and resource limitations may also challenge comprehensive alignment of medication and treatments goals with patients [17]. Thus, it can be difficult to meet patients where they are in relation to beliefs, wishes and values, moreover, involve patients actively in decisions about medication. In contrast, our study did not find an association between non-adherence and beliefs about necessity, concern,and harm, as reported in other studies. This might relate to differences across countries, languages and cultures, as described by Horne et al. [8].

Participants reported a high symptom burden and severity score consistent with previous HD studies [15, 32]. Higher symptom burden and severity were found to be significantly associated with non-adherence. Thus, similar to studies of patients living with HIV [9, 33]. Considering the impact of the symptom’s patient reported, it is perhaps not surprising that 32% of the participants exhibited non-adherent behavior. Difficulty concentrating, trouble sleeping and feeling tired are all symptoms that inadvertently could affect an individual’s ability to remember and adhere to complex dosing instructions. Additionally, dry mouth could easily hinder one’s ability to swallow pills, particularly for individuals’ who must adhere to strict fluid restrictions and at the same time take a substantial daily number of pills. Research has shown that patients with ESKD often experience symptom clusters that can impact related symptoms negatively [9, 16]. Zhou et al. describe five independent symptom clusters: gastro-intestinal discomfort, sleep disorder, skin discomfort and mood [16]. Nevertheless, regular symptom assessment lack standardization in dialysis settings, with limited treatment choices due to scarce evidence [32]. Moreover, research has identified a substantial discordance between patients experience of symptoms and those that are recognized by the HCP [32, 34]. Our results underscore the existing knowledge of the importance of HCPs to prioritize symptom management in this patient population. We propose incorporating validated patient reported outcome measures for routine screening and improving patient centered communication and care, aligning with recent KDIGO recommendation [32]. Exploring the potential of mobile applications to monitor and direct attention to symptoms during consultations has demonstrated promise in improving both symptom management and adherence to oral anticancer treatment [35]. Therefore, this area may be worth investigating in future research.

The strengths of this study include the multi-site design, the involvement of PRCs, the use of validated questionnaires and the relatively large sample size. However, a key limitation include the use of a cross-sectional design, which captures a moment, lack causality, thus affecting the generalizability of our study [36–38]. The choice of not including an assessment of depression and anxiety can also be seen as a limitation. Furthermore, the participants in our study were characterized by being older and having a higher degree of education, than those included in similar studies. Lastly, we did not conduct any analysis of patients who declined to participate.

In conclusion, the prevalence of non-adherence was 32% (95% CI = 27–37%). Patients’ beliefs about overuse were significantly associated with non-adherence, while patients’ beliefs about necessity, concern and harm were not. Moreover, symptom burden and severity were significantly associated with non-adherence with some of the most prominent symptoms being feeling tired/lack of energy, itching, dry mouth, lightheadedness/dizziness, trouble falling asleep/staying asleep and difficulties concentrating. Our results suggest HCP should prioritize discussions about medication adherence with patients, focus on addressing the patient-HCP relationship and patients’ symptom experience. Our results suggest HCPs should prioritize discussions about medication adherence with patients, focus on addressing the patient-HCP relationship and patients’ symptom experience. Future research is recommended to explore the effects on systematically using validated adherence measures in clinical practice on medication adherence, patient-HCP communication and trust. Furthermore, studies are warranted to delve deeper into the relationship between symptom burden and adherence in this population.

### Supplementary Information

**Additional file 1.** Supplementary material. Overview of analysis with variables and confounders.

## Data Availability

The datasets used and/or analyzed during the current study are available from the corresponding author on reasonable request.

## Abbreviations

BMQ: Beliefs about medicine questionnaire
CI: Confidence Interval
DSI: Dialysis symptom index
EORCT: European organization for research and treatment of cancer
ESKD: End stage kidney disease
HADS: Hospital anxiety and depression scale
HD: Hemodialysis
HCP: Health care professional
IBM SPSS: International business machine cooperation statistical package for the social sciences
IQR: Interquartile ranges
KDQOL: Kidney disease quality of life
MARS: Medication adherence report scale
OR: Odds ratio
PRC: Patient research consultant
SD: Standard diviation
