# Supplementary material for: Non-adherence, medication beliefs and symptom burden among patients receiving hemodialysis -a cross-sectional study
Source: BMC Nephrol. 2023 Oct 27;24:321. doi: 10.1186/s12882-023-03371-3 (PMC10604404; doi:10.1186/s12882-023-03371-3)
Supplement: Supplementary file 1 — Additional file 1. Supplementary material. Overview of analysis with variables and confounders. [file 12882_2023_3371_MOESM1_ESM.docx]

Supplementary material: Overview of analysis with variables and confounders

|  | **Dependent variable** | **Independent variable** | **Confounders** | **Change estimates** |
| --- | --- | --- | --- | --- |
| Analysis 1 | Non-adherence | Necessity | Symptom burden, Symptom severity, Longevity of hemodialysis, Country of birth, | -0.019 to +0.012 |
| Analysis 2 | Non-adherence | Concern | Symptom burden, Symptom severity, Longevity of hemodialysis | -0.022 to +0.022 |
| Analysis 3 | Non-adherence | Harm | Longevity of hemodialysis, Marital status, Living arrangement | +0.021 to +0.028 |
| Analysis 4 | Non-adherence | Overuse | Symptom burden, Marital status, Living arrangement | -0.012 to +0.021 |

|  | **Dependent variable** | **Independent variable** | **Confounders** | **Change estimates** |
| --- | --- | --- | --- | --- |
| Analysis 1 | Non-adherence | Symptom burden | Marital status, Living arrangement, Country of birth | +0.006 to +0.013 |
| Analysis 2 | Non-adherence | Symptom severity | Longevity of hemodialysis, Marital status, Living arrangement | -0.001 to +0.002 |
